# Synthetic half-reactions

**DOI:** 10.1039/d0sc03876h

**Published:** 2020-09-09

**Authors:** Andrei K. Yudin

**Affiliations:** Davenport Chemistry Laboratories, Chemistry Department, University of Toronto 80 St. George Street Toronto ON M5S 3H6 Canada andrei.yudin@utoronto.ca

## Abstract

This perspective on reactivity introduces Synthetic Half-Reactions (SHRs) as a way to analyze chemical transformations. SHRs denote either an uphill transformation leading to a higher energy state or a downhill transformation leading to a lower energy state. Using well-established processes, I show how the matching of different classes of SHRs offers a tool to classify chemical transformations. This raises the possibility to discover new processes by finding underappreciated combinations of endergonic and exergonic steps.

## Abstract 2

This perspective on reactivity introduces Synthetic Half-Reactions (SHRs) as a way to analyze chemical transformations.

## Graphical abstract

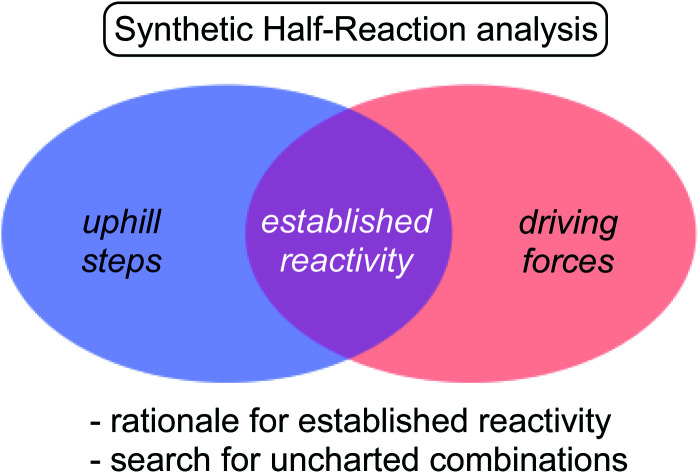

## Introduction

**Fig. 1:**
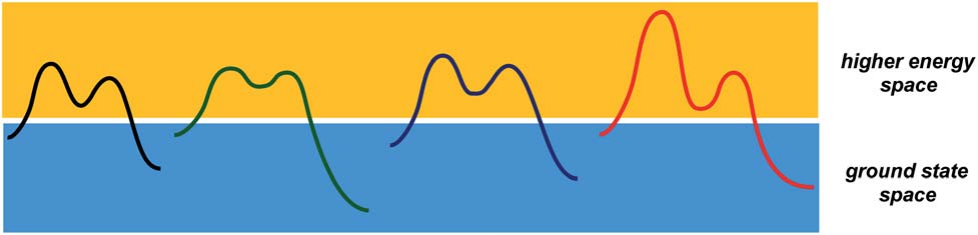
A schematic representation of the two conceptual domains of organic chemistry: the ground state space (blue rectangle) and the higher energy space (yellow rectangle).

While transition states and intermediates are central to our understanding of chemical transformations and their mechanisms, relatively stable structures that correspond to starting materials and products dominate efforts to formulate tangible objectives in chemical synthesis. What if, instead of focusing on starting materials and products, chemists had a formalism to dissect the structural correspondence between higher energy states that form in different processes (Fig. 1)? Any multistep reaction offers plenty of combinations between endergonic and exergonic steps. I would argue that the corresponding matches carry substantial pedagogical and practical potential for the discovery and understanding of chemical processes. But, even though chemistry papers routinely present evidence in support of metastable structures, there is currently no way to use accessible search engines in order to evaluate high energy states that correspond to reactive intermediates and unfavorable conformations. This is because intermediates are not being indexed by popular search engines. As part of the special collection on chemical reactivity, this perspective considers a way to analyze and catalog metastable states.

**Fig. 2:**
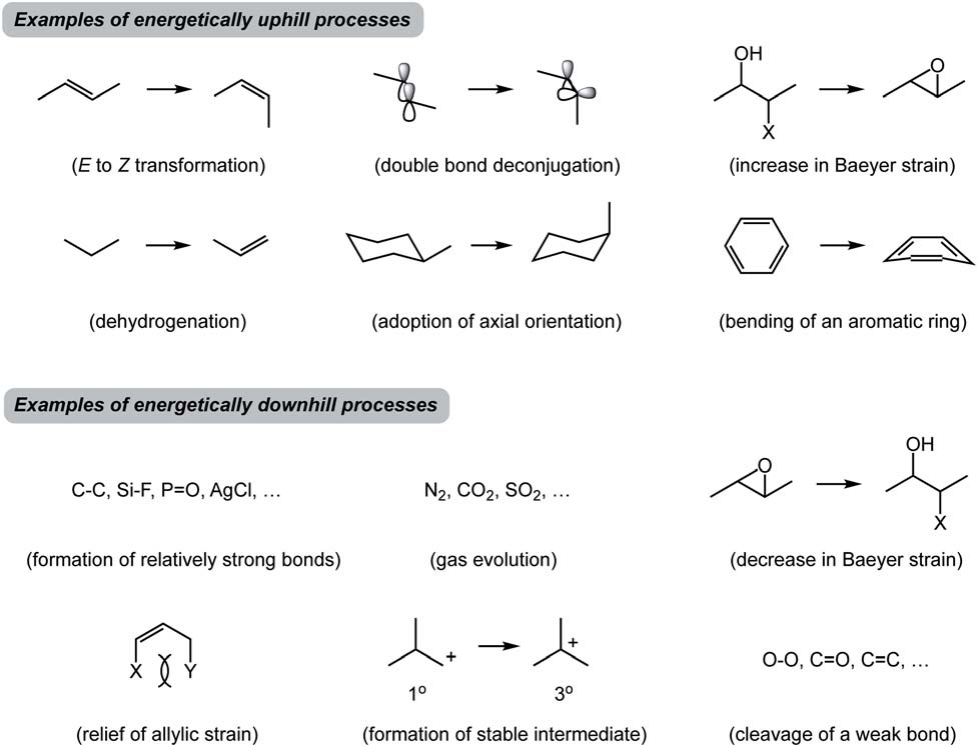
Representative examples of commonly encountered uphill and downhill processes.

Before I outline the proposed system of thought, a brief historical overview of metric-driven approaches is in order. Over the years, several quantitative and/or empirical formalisms have enhanced our understanding of chemical transformations. Thermochemical BDE (Bond Dissociation Energy) analysis is a useful tool to assess reaction thermodynamics, but it does not take into consideration the subtleties of molecular structures.^1^ Knowledge of acid dissociation constants is a classic example that allows one to use p*K*_a_'s of common functional groups in order to perform acid/base chemistry. However, application of p*K*_a_ towards understanding covalent bond formation is limited because basicity is not always an appropriate proxy for nucleophilicity.^2^ In this regard, Mayr's scale of nucleophilicity is a powerful tool for quantitative matching of reactive nucleophile/electrophile pairs.^3^ This approach is based on calculating rate constants from experimentally determined nucleophilicity and electrophilicity parameters, but it does not explicitly account for structural features of the molecules under consideration. Frontier molecular orbital analysis represents a treatment that allows one to match interacting orbitals of the reaction components.^4^ In practice, this analysis is primarily used to evaluate reactive π-rich systems and focuses on specific groups of atoms in molecules. It appears that there is no high energy state-driven formalism to determine productive combinations of thermodynamically favored and disfavored transformations. Although synthetic chemists are attuned to the concept of driving force (Fig. 2), it is not always straightforward to identify thermodynamically downhill reactions unless they are associated with well-recognized concepts such as formation of relatively strong bonds. It can also be difficult to recognize endergonic processes, which are invariable constituents of multistep reactions. That said, how might we dissect chemical processes into energetic components that may facilitate the design of new reactions and offer teachable qualities?

## Synthetic half-reactions

**Fig. 3:**
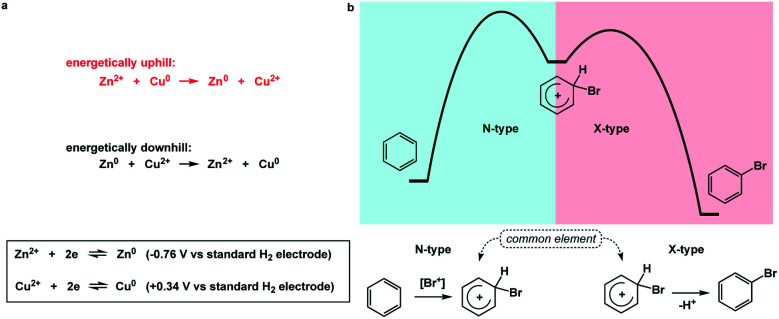
(a) The use of electrochemical half-reactions to evaluate the feasibility of metal ion reduction; (b) electrophilic aromatic substitution as an example of spatioenergetic matching between endergonic (N-type) and exergonic (X-type) processes.

I propose the term Synthetic Half-Reactions (or SHRs) to represent either an endergonic step that delivers a structure that is higher in energy than the starting point (N-type process) or an exergonic step that starts off a higher energy structure and leads to a more stable endpoint (X-type process). I would offer a loose analogy between SHRs and electrochemical half-reactions, which are commonly used to assess the thermodynamic feasibility of redox processes.^5^Fig. 3a features a Zn/Cu pair. Electrochemical half-reactions offer a powerful means to demonstrate that zinc oxidation provides the driving force for electron transfer to copper ions. Standard electrode potentials are used to demonstrate that zinc metal can easily reduce copper ions. On the other hand, the use of copper metal to reduce zinc ions is thermodynamically uphill. Taking inspiration from electrochemical half-reactions, consider the well-known electrophilic aromatic substitution. This process can be thought of as two energetic components (Fig. 3b). The first step is the thermodynamically uphill electrophilic addition (N-type), whereas the second step (X-type) is the driving force that involves proton elimination and concomitant restoration of aromaticity. Upon comparison of this pair of N- and X-processes, it is easy to see that they are ‘matched’ because sigma complex acts as the common element. I will refer to such instances as spatioenergetic matches in order to suggest that, for a given reaction to succeed, the energetic benefits of the driving force should ideally operate within the footprint of the endergonic step. The idea of footprint is intuitive: chemical transformations are associated with a well-defined three-dimensional space (footprint) where a given process operates. Obviously, in the case of electrophilic aromatic substitution, the endpoint of the N-type half-reaction and the starting point of the X-type half-reaction are structurally superimposable, *i.e.* they operate within the same footprint. If the entire process – the SEAr reaction in this instance – were not known, but information on the SHRs mentioned above was available, it would be straightforward to envision the electrophilic aromatic substitution process. It follows that one way to understand chemical reactivity might be to find underexplored matches between endergonic SHRs and spatioenergetically appropriate exergonic SHRs. A related objective is to evaluate instances in which interacting components are matched in several distinctly different ways. This would eventually allow one to understand how to design successful reactions from overlapping N- and X-combinations that are yet to be considered.

**Fig. 4:**
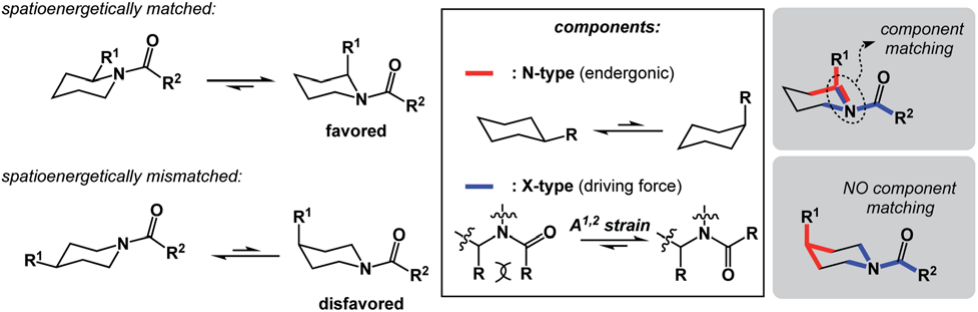
Allylic strain relief matched with an equatorial-to-axial transition in acylated piperidine.

In addition to chemical transformations, wherein interatom connectivity is altered by way of covalent bond formation and cleavage, conformational transitions can also be dissected using this method and/or matched with covalent bond-forming steps. To illustrate the importance of correct spatioenergetic matching, consider an allylic strain-driven solution to creating molecules with axial substituents on the piperidine ring.^6^ Here, placement of the R^1^ group in the axial position is the energetically uphill N-type SHR (shown in red in Fig. 4), whereas minimization of the steric repulsion between R^1^ and R^2^ groups is the X-type SHR (shown in blue in Fig. 4). The top equilibrium shown in Fig. 4 is favored because N- and X-components are spatioenergetically matched. As a result, one could say that the thermodynamic benefits of allylic strain relief allow for equatorial-to-axial transition of the R^1^ substituent because there is a two-atom SHR overlap. This process can be appropriately termed component matching. If N- and X-processes were separated by more atoms (*e.g.* the bottom example in Fig. 4) and the goal were to achieve the axial orientation of the R^1^ substituent, component matching would not be possible and the R^1^ group would prefer to reside in the equatorial position.

**Fig. 5:**
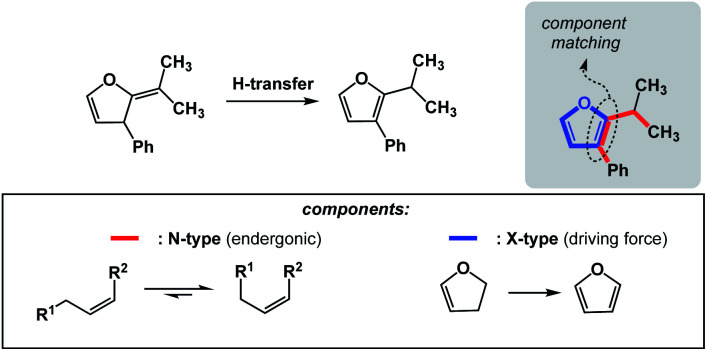
Aromaticity-driven relief of allylic strain.

The requirements for spatioenergetic matching are not complicated and should make intuitive sense. For instance, one can anticipate failures when energetic benefits accrued in an X-type component are not sufficient to account for the endergonic nature of an N-type component, even when the two are matched in an appropriate way. For example, an almost certain failure would arise from matching allylic strain release (common energy gain: 3–5 kcal mol^−1^)^7^ with disruption of aromaticity (common cost: 40–60 kcal mol^−1^).^8^ Of course, the opposite scenario would be straightforward. Fig. 5 shows how aromaticity can alleviate the endergonic build-up of allylic strain.^9^ In this case, the developing aromatic system of furan is the driving force for the release of the allylic strain in the starting material.

**Fig. 6:**
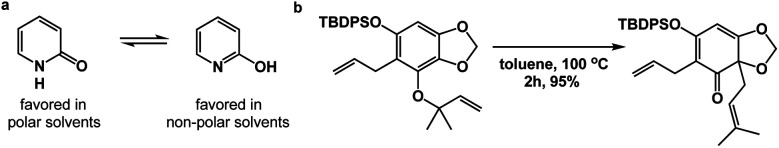
The use of carbonyl group-based driving forces to disrupt aromaticity: (a) pyridine/pyridone system; (b) the use of Claisen rearrangement to disrupt aromaticity.

Spatioenergetic matching should heavily rely on common functional groups that are associated with broad reactivity profiles. Thus, the carbonyl group could be seen as a versatile control element during this process. One can formulate unusual and interesting questions such as: can a carbonyl-based functionality serve as a means to disrupt aromaticity? Fig. 6a illustrates the solvent-dependent tautomerization in the pyridine/pyridone system. Amide bond formation, depending on the medium, can override pyridine aromaticity in this reversible process. Granted, this example has limited value because no skeletal change is taking place. On the other hand, the reaction featured in Fig. 6b showcases a Claisen rearrangement that disrupts aromaticity with concomitant formation of the γ,δ-unsaturated carbonyl unit, which should be rightfully associated with considerable synthetic utility.^10^

**Fig. 7:**
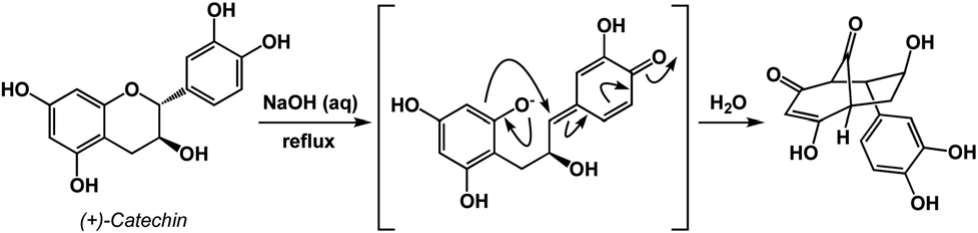
A retro-conjugate addition in the context of carbonyl-based driving forces.

An instructive transformation that features carbonyl-driven dearomatization is shown in Fig. 7.^11^ The pivotal intermediate in this example is formed by retro-conjugate addition, which introduces reverse processes as an exciting feature relevant to spatioenergetic analysis. Upon C–O bond scission, the enolate here acts as the C-nucleophile and leads to the disruption of aromaticity with concomitant formation of two carbonyl groups. Context-dependent deployment of microscopic reverses of known processes might represent a particularly interesting utility of spatioenergetic analysis.

**Fig. 8:**
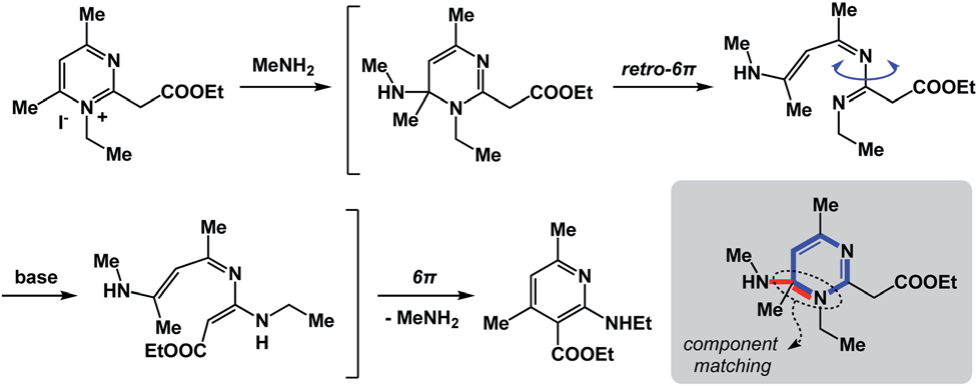
The “cross-talk” between nucleophilic addition and electrocyclization.

The fundamental distinction of the proposed approach is to be accommodating of as many distinct reactivity types as possible. The sole requirement is for the N-type process to be spatioenergetically matched with its X-type counterpart. An examination of complex mechanistic pathways supports the feasibility of matching mechanistically distinct reaction classes. For instance, in the Kost–Sagitullin rearrangement (Fig. 8) the nucleophilic addition leads to the formation of a tetrahedral intermediate that is spatioenergetically matched with retro-electrocyclization.^12^ This process is followed by bond rotation, tautomerization, electrocyclization, and amine elimination to generate the pyridine core. I hypothesize that more cases of this kind could be predicted if there was a way to exhaustively evaluate a wide range of SHR combinations. A searchable algorithm to accomplish this goal would go a long way in complementing the existing search engine capabilities.

**Fig. 9:**
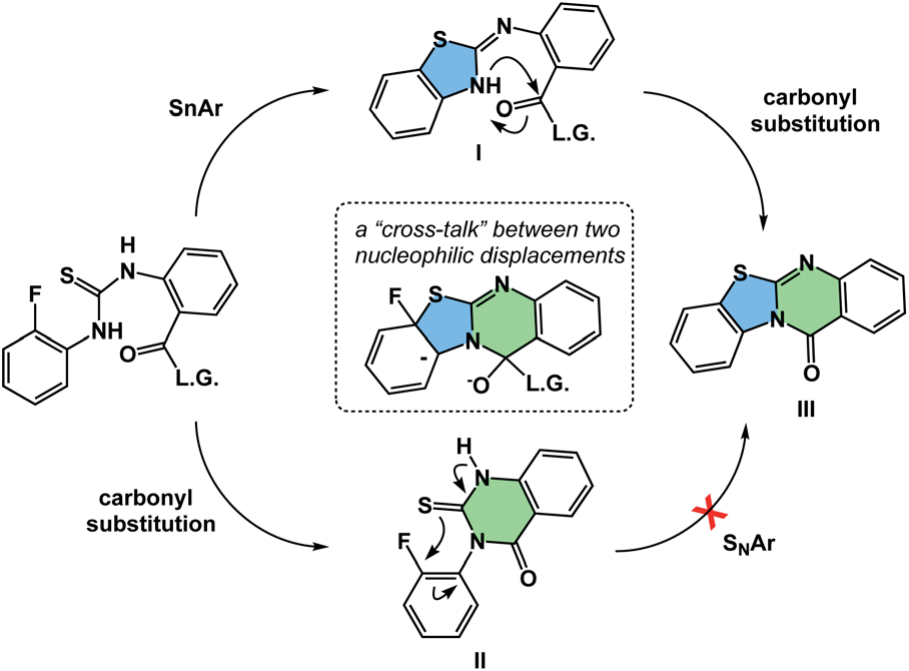
Addressing the question of sequence in spatioenergetic matching.

The process of matching sequential transformations can be far from straightforward and underscores the enduring challenges of organic chemistry. Let's consider the simple instance of nucleophilic aromatic substitution and amine acylation operating in a cascade fashion.^13^ The process shown in Fig. 9 traverses several barriers, but the mechanistic sequence matters because compound **II**, made as a control, does not undergo tetracycle formation. On the other hand, the initial 5-membered ring formation appears to be on the right track, leading to the final product **III***via* intermediacy of **I**. Understanding factors that control the behavior observed in such cases could facilitate the design of hybrid processes.

**Fig. 10:**
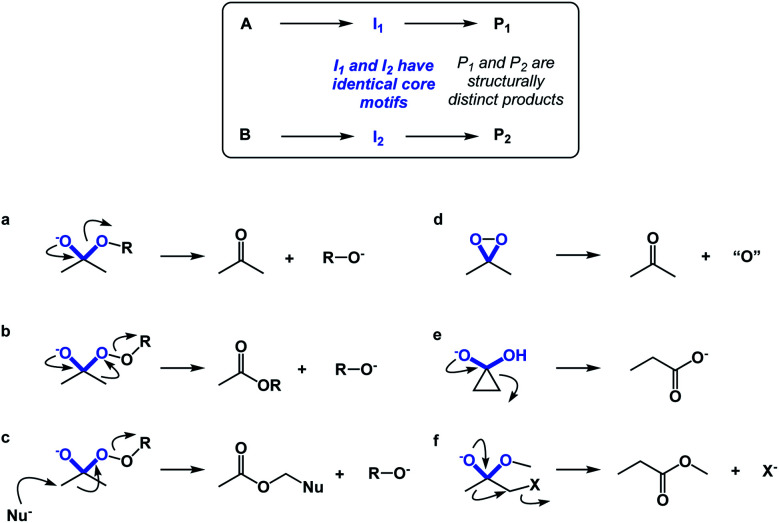
Core motif analysis as a means to illustrate the divergence of pathways: (a) collapse of the tetrahedral intermediate during ester hydrolysis; (b) the Baeyer–Villiger pathway; (c) nucleophilic attack with concomitant O–O bond scission; (c) oxygen atom transfer; (d) Favorskii rearrangement; (e) semipinacol pathway.

If spatioenergetic matching is a potentially viable approach, it should be possible to speculate that relatively simple substructures can support a variety of reaction mechanisms in a context-dependent manner. To substantiate this point, I would perform a straightforward core motif analysis. For the purposes of this paper, a core motif is defined as atoms in the immediate proximity to the atoms that undergo chemical transformation. It can be shown that even seemingly trivial core motifs are associated with widely differentiated and context-dependent outcomes. As a representative core motif, I would offer the sp^3^ carbon in the ketone oxidation state. Ordinarily, the tetrahedral intermediate that features O–C–O core collapses to generate the carbonyl group (Fig. 10a). However, appending a weak O–O bond to the periphery of the O–C–O core dramatically remodels the accessible energy landscape, leading to ester formation through carbon-to-oxygen migration (the Baeyer–Villiger pathway, Fig. 10b). In the presence of a nucleophile, the reaction course changes yet again, despite the fact that the outcome is still driven by O–O bond scission.^14^ On the other hand, if the core motif is maintained, but oxygen atoms are connected to each other, atom transfer pathway starts to dominate (Fig. 10d), while if the α-carbon atoms are joined through a C–C bond, the Favorskii rearrangement becomes the dominant pathway (Fig. 10e). Lastly, the presence of a leaving group at the alpha position gives rise to a semipinacol-like reactivity^15^ (Fig. 10f). Taken together, the multitude of bond-breaking and bond-making events that unfold in structures that feature the relatively simple O–C–O core reflects the effect of driving force variation and highlights the nuances of spatioenergetic matching in chemistry.

## Conclusions

In closing, traditional training in synthetic chemistry encourages us to seek familiar sub-structures in target molecules and recognize how they could be built using appropriate inputs that correspond to stable molecules with established reactivity. While seeking such patterns will undoubtedly continue to drive innovation, there might be benefits to a complementary tool that lowers our reliance on structural similarity between starting materials in favor of spatioenergetic matching that involves higher energy states. My hypothesis is that it should be possible to discover new reactions by understanding how well-known endergonic SHRs can be spatioenergetically matched with established exergonic ones in previously underappreciated ways. Search engines such as Reaxys or SciFinder do not offer an opportunity to evaluate higher energy states. The examples described above arise from the SHR treatment of known reactions, but another interesting goal would be to design entirely new transformations using spatioenergetic matching. The central hypothesis is that one should be able to synergize SHRs that have not yet been spatioenergetically matched. There are many well-known N- and X-type processes that are familiar to an organic chemist (Fig. 2). While a plethora of N/X combinations correspond to well-established processes, it is reasonable to suspect that there will be productive combinations that have not received prior attention.

## Conflicts of interest

There are no conflicts to declare.
